# Comparison of Two Assays to Determine Anti-Citrullinated Peptide Antibodies in Rheumatoid Arthritis in relation to Other Chronic Inflammatory Rheumatic Diseases: Assaying Anti-Modified Citrullinated Vimentin Antibodies Adds Value to Second-Generation Anti-Citrullinated Cyclic Peptides Testing

**DOI:** 10.1155/2014/198198

**Published:** 2014-06-15

**Authors:** Miriam Lizette Díaz-Toscano, Eva Maria Olivas-Flores, Soraya Amali Zavaleta-Muñiz, Jorge Ivan Gamez-Nava, Ernesto German Cardona-Muñoz, Manuel Ponce-Guarneros, Uriel Castro-Contreras, Arnulfo Nava, Mario Salazar-Paramo, Alfredo Celis, Nicte Selene Fajardo-Robledo, Esther Guadalupe Corona-Sanchez, Laura Gonzalez-Lopez

**Affiliations:** ^1^Programas de Doctorado en Farmacología y en Ciencias Biomédicas, Centro Universitario de Ciencias de la Salud (CUCS), Universidad de Guadalajara, Guadalajara, JAL, Mexico; ^2^Hospital General Regional 180 IMSS Tlajomulco, JAL, Mexico; ^3^Unidad de Investigación en Epidemiología Clínica y División de Investigación en Salud, UMAE, Hospital de Especialidades (HE), Centro Médico Nacional de Occidente (CMNO), IMSS, Guadalajara, JAL, Mexico; ^4^Programa de Doctorado en Salud Púbica CUCS, Guadalajara, JAL, Mexico; ^5^Departamento de Fisiologia, CUCS, Universidad de Guadalajara, Guadalajara, JAL, Mexico; ^6^Programa de Servicio Social en Investigación, Secretaria de Salud, Guadalajara, JAL, Mexico; ^7^Decanato Ciencias de la Salud, Universidad Autónoma de Guadalajara, Mexico; ^8^Dirección de la División de Disciplinas para el Desarrollo, Promoción y Preservación de la Salud, CUCS, Universidad de Guadalajara, Mexico; ^9^Departamento de Medicina Interna-Reumatología, Hospital General Regional 110, Instituto Mexicano del Seguro Social (IMSS), Guadalajara, JAL, Mexico; ^10^Avenida Salto del Agua 2192, Colonia Jardines del Country, Guadalajara, Mexico

## Abstract

Determination of anti-citrullinated peptide antibodies (ACPA) plays a relevant role in the diagnosis of rheumatoid arthritis (RA). To date, it is still unclear if the use of several tests for these autoantibodies in the same patient offers additional value as compared to performing only one test. Therefore, we evaluated the performance of using two assays for ACPA: second-generation anti-citrullinated cyclic peptides antibodies (anti-CCP2) and anti-mutated citrullinated vimentin (anti-MCV) antibodies for the diagnosis of RA. We compared three groups: RA (*n* = 142), chronic inflammatory disease (CIRD, *n* = 86), and clinically healthy subjects (CHS, *n* = 56) to evaluate sensitivity, specificity, predictive values, and likelihood ratios (LR) of these two assays for the presence of RA. A lower frequency of positivity for anti-CCP2 was found in RA (66.2%) as compared with anti-MCV (81.0%). When comparing RA versus other CIRD, sensitivity increased when both assays were performed. This strategy of testing both assays had high specificity and LR+. We conclude that adding the assay of anti-MCV antibodies to the determination of anti-CCP2 increases the sensitivity for detecting seropositive RA. Therefore, we propose the use of both assays in the initial screening of RA in longitudinal studies, including early onset of undifferentiated arthritis.

## 1. Introduction 

Rheumatoid arthritis (RA) is a chronic inflammatory disorder that involves synovial joints and may develop extra-articular manifestations [1]. Frequently, the diagnosis of RA may pose some difficulties in primary care, particularly during early disease, and this disease may inappropriately be confused with other rheumatic diseases [2]. In this context, a relevant tool to support the diagnosis is the presence of autoantibodies associated with the disease. Although the detection of rheumatoid factor [3] is useful to support the diagnosis and it is detected in 75% of patients with RA, a limitation of this autoantibody is its low specificity, being frequently observed in other rheumatic disorders, chronic infections, and even in healthy elderly people [3]. Different assays are currently used to detect antibodies against cyclic citrullinated antigens as well as noncyclic citrullinated peptides. Therefore, the term anti-citrullinated peptide antibody (ACPA) is commonly used in these days. Assays to identify antibodies against citrullinated cyclic peptides are commonly used as a tool to support the diagnosis of RA, because it has been widely demonstrated that these autoantibodies have higher specificity as compared with the rheumatoid factor (RF). One of the most common assays is the determination of second-generation anti-citrullinated cyclic peptide antibodies (anti-CCP2). Therefore, ACPAs have been included in the most recent classification criteria for RA diagnosis [4]. Nevertheless, around 38% of patients with RA may have negative results for anti-CCP2 [5, 6].

Assays determining antibodies against human mutated vimentin (anti-MCV) have been also proposed recently as a tool for the diagnosis of RA [7, 8]. Nevertheless, still 26% of patients with RA may yield negative results with these assays [7]. To date, there are several studies comparing the performance of different assays of anti-CCP2 versus anti-VCM in the diagnosis of RA [9–11]. These studies support that detection of anti-VCM is as useful as the assays determining anti-CCP2 to distinguish RA from healthy controls [12, 13] and can help in the differential diagnosis of RA from other rheumatic disorders [14–16]. Nevertheless, currently, there are no studies in Mexican patients evaluating if the strategy of performing both tests may increase sensitivity and positive predictive value for the presence of established RA as compared to performing them individually.

Therefore, we evaluated the performance of using two ACPA assays: second-generation anti-citrullinated cyclic peptide antibodies (anti-CCP2) and anti-mutated citrullinated vimentin (anti-MCV) antibodies in established RA, and we correlated the titers observed of these autoantibodies with disease activity.

## 2. Patients Methods

*Design*. Cross-sectional study.

*Clinical Setting*. Adult consecutive patients with RA seen in an outpatient rheumatology clinic of a secondary-care center in Guadalajara, Mexico (*Hospital General Regional 110, Instituto Mexicano del Seguro Social*), were invited to participate if they met at least four of the 1987 ACR criteria for RA [17]. They were excluded if they had a history of blood transfusion, chronic infectious diseases, including hepatitis B or C, human immunodeficiency virus, or tuberculosis. Patients with overlapping syndrome, cancer, or other associated autoimmune disorders or pregnant patients were also excluded.

These patients were compared with two distinct non-RA controls selected.

(i) The first comparison group was constituted by patients with other rheumatic inflammatory disorders mainly including systemic lupus erythematosus (SLE, 1982 ACR criteria) [18] or ankylosing spondylitis (AS, 1984 New York modified criteria) [19]. Nevertheless, patients with systemic sclerosis (SSc) and articular manifestations were included if they met the 1980 ACR criteria [20]. All these patients were obtained from the same rheumatology clinic where patients with RA were recruited.

(ii) The second group was constituted by clinically healthy blood donors obtained from the same hospital, without history of blood transfusion or chronic infections.

For these two comparison groups, similar inclusion and exclusion criteria described for patients with RA were applied.

### 2.1. Clinical Evaluations

A structured assessment for patients with RA was performed including disease characteristics, evaluation of disease activity according to DAS-28 [21], functioning according to the Spanish validated version of HAQ-DI [22], and treatments used.

### 2.2. ACPA Determinations

A venous blood sample was taken from all included subjects at the same time of the clinical evaluation and the serum was obtained and stored at −20°C until antibodies determination. Anti-CCP2 were determined by ELISA using a commercial kit (Axis-Shield, UK) with a cut-off value for positivity >5 U/mL and anti-MCV were determined by ELISA using also a commercial kit (ORGENTEC, Mainz, Germany) with a cut-off value for positivity >20 U/mL.

## 3. Statistical Analysis 

Qualitative variables were expressed as frequencies and percentages and quantitative variables were expressed as means ± standard deviations. Chi-square tests were used to compare proportions among groups and Student's *t*-test was used to compare means between two groups. We selected as “gold standard” the 1987-ACR criteria for diagnosis of established RA. These criteria were used instead of the most recent 2010-ACR criteria because the status of positive ACPA is included within the criteria. The performance of the assays for anti-MCV and anti-CCP2, either individually or tested together, to identify RA was evaluated estimating sensitivity, specificity, and positive and negative predictive values, as well as likelihood ratios. In this study, sensitivity can be defined as the probability of positive anti-CCP2 or anti-MCV in patients with RA. Specificity was defined as the probability of negative results for these autoantibodies in patients or controls without RA. Positive predictive value (PPV+) was defined as the probability of having RA in presence of anti-CCP2 or anti-MCV. Negative predictive value was defined as the probability of not having RA in presence of a negative result for these autoantibodies. We computed 95% confidence intervals (95% CI) for the utility values for these autoantibodies. Kappa statistics was used to compute the degree of agreement in positivity between both anti-CCP2 and anti-MCV for patients with RA.

Correlation between titers of anti-CCP2 and anti-MCV and variables was examined using Spearman's correlation coefficient. The value of statistical significance was set at a *P* value of <0.05. All analyses were done with the SPSS program (version 8).

## 4. Results

One hundred and forty-two patients with RA were included and compared with 86 patients in the group of autoimmune rheumatic diseases (33 with SLE, 44 with AS, and 9 with SSc) and 56 healthy controls.

General characteristics of patients with RA are shown in Table 1. Additional data, not shown in this table, include that 83% of patients with RA had an active disease (DAS-28 index >3.2) and 26.6% had a significant degree of disability (HAQ-DI > 1.25). At the time of the evaluation, most of the patient received glucocorticoids, 56 patients (76%) used a dose of ≤5 mg, which is considered a low dose.

Concordance between the findings of the two assays, anti-CCP2 and anti- MCV, in RA is shown in Table 2. Only around 62% of the patients showed positivity for both assays, anti-CCP2 and anti-MCV, allowing for a Kappa = 0.42 value for Kappa statistics.

An evaluation of utility values for the strategies of testing each assay, anti-CCP2 or anti-MCV alone, or testing both assays in established RA compared with clinically healthy blood donors is shown in Table 3. The highest sensitivity was observed when both autoantibodies tests were performed (85%) followed by testing anti-MCV alone (81%), whereas the lowest sensitivity was observed when only anti-CCP2 test was performed. On the other side, specificity and PPV(+) were similar with the three strategies, and the NPV(−) increased substantially, if both assays were negative.

The utility values for the strategies of performing only anti-CCP2 or anti-MCV or both of these assays in established RA compared with other rheumatic inflammatory diseases are shown in Table 4. The highest sensitivity was again observed when both assays were performed (85%) and the lowest sensitivity was attained when using only anti-CCP2 (66%). The highest specificity was observed when only anti-VCM was performed (96%). PPV(+) values were higher with the anti-MCV assay alone (97%), whereas the highest NPV(−) was observed when both assays were negative (79%).

## 5. Discussion

In our study, we observed that the assay for anti-MCV antibodies showed more sensitivity and specificity than the assay for anti-CCP2 antibodies to distinguish established RA patients from other systemic inflammatory rheumatic diseases. Using the strategy of performing both assays, we obtained an increase in sensitivity in comparison with using either assay individually. In our study, the Kappa between both assays indicates that determination of both tests should be complementary and consequently increases the utility of both tests in the clinical armamentarium without decreasing specificity.

Previous studies have reported, for anti-CCP2, specificities greater than 90% [23–25], similar to our findings where we found a specificity of 92% for CIRD and 94% for CHS, this assay being very useful to exclude people who do not have RA.

Nevertheless, in terms of a screening test, a higher sensitivity is extremely relevant; therefore, strategies to increase the values of sensitivity are required to establish an earlier diagnosis and opportune reference to the rheumatologist. To this regard, in the present study, the utilization of an assay for anti-CCP2 exclusively had only 66% of sensitivity, whereas when both assays, anti-CCP2 and anti-MVC, were done in the same patients, the sensitivity increased to 85%, with an improvement in the utility of these assays as a tool for clinicians. Regarding specificity of anti-CCP2, some studies have shown a wide variability ranging from 40% to 83% [26, 27], the frequency of negatives being a limitation to establish the diagnosis in RA. Genetic factors may contribute to these differences in sensitivity, characteristic of the study population, including variables such as disease duration or severity of the disease, and characteristics of assays used to detect these autoantibodies [28], although, in our study, anti-MCV antibodies were more sensitive than anti-CCP2 antibodies for RA and these findings have been reported by others [29]. To this regard, around 1 of 5 patients with established RA had a negative anti-MCV test result. Therefore, the question arises if the utility value of the test could be increased by using both assays. We observed that using both assays in the same patients the sensitivity increases to 85% with an LR+ of 10.47 in comparison to other CIRD, constituting an excellent support in the clinical armamentarium for RA.

Several factors could contribute to explaining why we observed that the anti-VCM assay was more sensitive than the anti-CCP2 assay. One of them is that vimentin contains 43 arginine residues. Each arginine residue can potentially be citrullinated by peptidylarginine deiminase (PAD) resulting in a variety of citrullinated epitopes. In contrast, in the anti-CCP2 test only a few epitopes are presented [30–32].

Some authors reported recently that combining determinations of anti-MCV, anti-CCP2, and RF increases the sensitivity [15]. Nicaise-Roland et al. [29] described, in a cohort of patients with early RA and undifferentiated arthritis, an increase in sensitivity when two tests are associated. Therefore, these data support our findings implying gains in clinical utility when two assays for ACPA are applied in the same patient. Our study, however, revealed that still 6% of controls without any rheumatic disorders had positive anti-CCP2 or anti-MCV antibodies; these data are relevant because the presence of a positive antibody without clinical manifestations is insufficient to support the presence of disease, although we ignore it if these patients would have an increase in risk for a CIRD in the future. Cohort studies will help to identify the evolution of these patients with positive anti-MCV.

Some limitations of the study due to its cross-sectional nature are that we were unable to identify if controls without rheumatic disorders who depicted positivity to one or both autoantibodies will have progression to a CIRD in the future; nevertheless, this hypothesis should be tested in cohort models, increasing the number of patients. On the other side, we did not apply these tests to specific subgroups of patients, such as RA with extra-articular manifestations, undifferentiated arthritis, or early RA, where the performance of these diagnostic tests may have substantial variations to those observed in defined RA. Another limitation was that we did not include an assay for testing anti-CCP3. Anti-CCP3 assays rely upon additional epitopes not present in the anti-CCP2 antigen sequence [33, 34]. Szekanecz et al. evaluated the sensitivity of cyclic citrullinated antibodies second-generation (anti-CCP2) and third generation (anti-CCP3 and anti-CCP3.1); the diagnostic sensitivity of anti-CCP2 was 74.8%, anti-CCP3 was 78.8%, and anti-CCP3.1 was 83.0%; the specificity of anti-CCP2 was 95.7%, anti-CCP3 was 96.6%, and anti-CCP3.1 98.3% [35]. However, Shidara et al. show no evident increase in utility values when comparing anti-CCP3 and anti-CCP2 assays; the sensitivity of anti-CCP2 was 88.7% and specificity of anti-CCP2 was 89.5%, whereas; the sensitivity of anti-CCP3 was 91.5% and specificity was 87.7% [36]. An assay for anti-CCP3 may provide an increase in sensitivity as compared to that observed with the assay for anti-CCP2 used in this study.

In conclusion, using both assays, anti-CCP2 and anti-MCV, increases the sensitivity for the presence of RA as compared to performing only one assay; therefore, this strategy should be included in the clinical armamentarium to improve the value of these assays as screening test.

## Figures and Tables

**Table 1:** General characteristics in patients with rheumatoid arthritis.

Characteristics	*N* = 142
Age in years, mean ± SD	49 ± 10.69
Women, *n* (%)	135 (95)
Disease duration (years), mean ± DE	9 ± 8.07
DAS-28, mean ± SD	4.7 ± 1.5
DAS-28 > 3.2 *n* (%)	118 (83.1)
HAQ-DI, mean ± SD	0.91 ± 0.65
HAQ-DI > 1.25, *n* (%)	38 (26.6)
Treatments	
Methotrexate, *n* (%)	48 (33.8)
Chloroquine, *n* (%)	4 (2.8)
Leflunomide, *n* (%)	17 (12)
Azathioprine, *n* (%)	18 (14)
Etanercept, *n* (%)	8 (5.6)
Glucocorticoids, *n* (%)	124 (87.9)
Prednisone mg, mean ± SD	5.7 ± 1.6

SD: standard deviation, mg: milligrams.

DAS-28: *disease activity score*.

DAS-28: low activity ≤3.2; moderate activity >3.2 y ≤ 5.1; high activity >5.1.

HAQ-DI: Health Assessment Questionnaire-Disability Index: S.

**Table 2:** Concordance between the results of assays for anti-CCP2 and anti-MCV in rheumatoid arthritis.

		Anti-CCP2	
		Positive *n* = 94 (66.2%)	Negative *n* = 48 (33.8%)
Anti-MCV	Positive		
	*n* = 115 (81.0%)	88 (61.97%)	27 (19.01%)
	Negative		
	*n* = 27 (19.0%)	6 (4.22%)	21 (14.78%)

Total patients with RA assessed = 142, and values in parenthesis represent the percentage of the total 142 patients.

Kappa = 0.42.

**Table 3:** Utility values of anti-CCP2, anti-MCV, or any of these assays in rheumatoid arthritis in comparison with clinically healthy subjects (CHS).

Utility values of the assays for anti-CCP2 and anti-MCV results	Anti-CCP2	Anti-MCV	Anti-CCP2 or anti-MCV
Sensitivity % (95% CI)	66 (58–74)	81 (73–87)	85 (78–91)
Specificity % (95% CI)	94 (84–99)	94 (84–99)	94 (84–99)
Positive predictive value % (95% CI)	97 (91–99)	97 (93–99)	97 (93–99)
Negative predictive value % (95% CI)	51 (41–61)	65 (53–75)	70 (58–81)
LR+	11.69 (3.87–35.32)	14.31 (4.75–43.07)	15.05 (5–45.28)
LR−	0.36 (0.28–0.46)	0.20 (0.14–0.28)	0.16 (0.11–0.23)
Prevalence	73 (66–79)	73 (66–79)	73 (66–79)

LR+: positive likelihood ratio; LR−: negative likelihood ratio.

**Table 4:** Utility values of anti-CCP2, anti-MCV, or any of these assays in rheumatoid arthritis in comparison with other chronic inflammatory rheumatic diseases (CIRD).

Utility values of the assays for anti-CCP2 and anti-MCV results	Anti-CCP2	Anti-MCV	Anti-CCP2 or Anti-MCV
Sensitivity % (95% CI)	66 (58–74)	81 (73–87)	85 (78–90)
Specificity % (95% CI)	92 (84–97)	96 (90–99)	92 (84–97)
Positive predictive value % (95% CI)	93 (86–97)	97 (93–99)	94 (89–98)
Negative predictive value % (95% CI)	62 (53–71)	75 (66–83)	79 (70–86)
LR+	8.13 (3.96–16.7)	23.22 (7.62–70.77)	10.47 (5.13–21.36)
LR−	0.37 (0.29–0.47)	0.20 (0.14–0.28)	0.16 (0.11–0.24)
Prevalence	62 (67–89)	62 (68–89)	62 (56–68)

LR+: positive likelihood ratio; LR−: negative likelihood ratio.
